# Resveratrol supplementation improved antioxidant capacity and mitigated the impact of heat stress in lactating dairy cows

**DOI:** 10.3168/jdsc.2025-0990

**Published:** 2026-02-19

**Authors:** M.R.R. Nair, Z. Yu, J.M. Cantet, M.S. Hasan, K. Frady, A.G. Ríus

**Affiliations:** Department of Animal Science, University of Tennessee Institute of Agriculture, Knoxville, TN, 37996

## Abstract

The relationship between oxidative stress, inflammation, and productivity loss associated with heat stress has been documented in dairy cattle. Resveratrol, a naturally occurring plant-based polyphenolic compound, possesses antioxidant properties because of its chemical structure. We hypothesize that supplementing resveratrol would improve the antioxidant capacity and reduce heat stress–induced oxidative stress in lactating dairy cows. The objective of the study was to determine the effects of resveratrol supplementation on plasma markers of oxidative stress and their relation with productivity, respiratory rate (RR), and rectal temperature (RT) in heat-stressed dairy cows. The study was conducted during summer using 30 Holstein cows averaging (±SD) 127 ± 33 DIM, 48.9 ± 8.9 kg/d milk yield, and 2.1 ± 1.0 parity housed in a sand-bedded freestall barn at the University of Tennessee dairy farm. Following a 1-wk baseline period, cows were allocated to 1 of 2 treatment groups, control (CON; n = 15, TMR with no resveratrol) or resveratrol (RES; n = 15, TMR with 0.5 g resveratrol/cow per day) for 2 wk of adaptation period. During baseline and adaptation periods, fans and soakers enhanced cows' evaporative cooling. Following the adaptation period, fans and soakers were disabled, cows experienced natural summer heat stress, and treatments were imposed for 3 wk. Temperature and humidity loggers recorded weather variables in the barn. Rectal temperature and RR were recorded 3 times daily. Blood samples were collected once a week, and plasma was isolated to measure markers of oxidative stress. Data were analyzed using the MIXED procedure with repeated measurements in SAS. The effects of treatment, week, parity, and their interactions were considered fixed, and animal was considered a random effect. Baseline measurements were included in the statistical analysis as covariate if significant. A suitable covariance structure was used based on the lowest Akaike and Bayesian information criteria. Regression analysis determined the relations between milk yield and plasma total antioxidant capacity, and RT or RR with plasma total antioxidant capacity. Results showed that the ambient temperature and relative humidity ranged between 20°C and 30°C and 49%–84% during the treatment period. Compared with CON, the RES treatment increased plasma total antioxidant capacity up to 12% in parity 2 cows. The RES treatment did not affect plasma concentrations of superoxide dismutase and malondialdehyde but tended to reduce plasma pro-oxidant–antioxidant balance in wk 2 and 3. Regression analysis indicated that plasma total antioxidant capacity was positively related to milk yield in both RES (R^2^ = 0.13) and CON cows (R^2^ = 0.02). Specifically, a 1 m*M* increase in plasma total antioxidant capacity was associated with an 8.5 kg higher milk yield in RES cows compared with CON cows. Regression analysis indicated that RR was explained weakly by plasma total antioxidant capacity in CON cows only (R^2^ = 0.01). In conclusion, RES increased plasma total antioxidant capacity and mitigated oxidative stress and production loss in heat-stressed dairy cows. Resveratrol could be used as a feed additive to reduce the effect of heat stress on commercial dairy farms.

## Highlights

• This study examined heat stress-induced oxidative stress and productivity loss in cattle.
• The study evaluated the effects of dietary supplementation of resveratrol on the oxidative status in heat stressed dairy cows.
• Supplementing resveratrol enhanced plasma total antioxidant capacity to mitigate oxidative stress in heat-stressed dairy cows.
• Increasing plasma antioxidant capacity had a positive relationship with milk yield in resveratrol cows.

## Graphical Abstract

**Figure:**
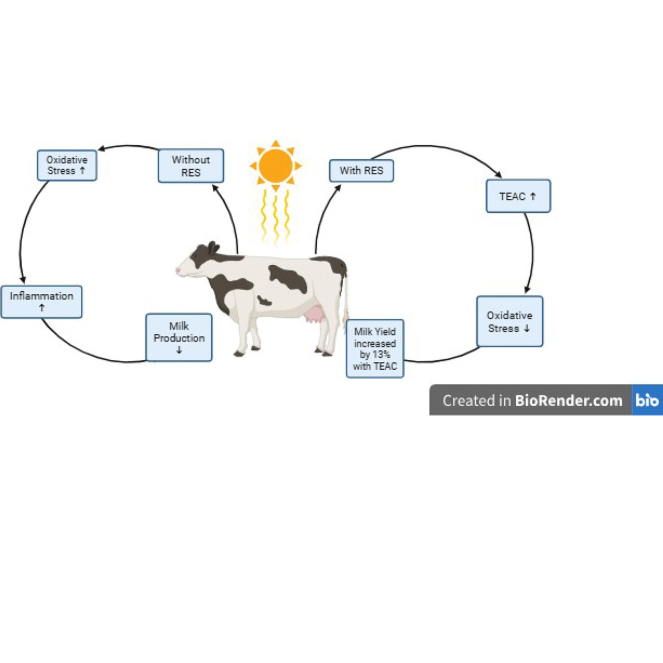
**Summary:** The objective of this study was to determine the effects of resveratrol supplementation on plasma markers of oxidative stress and their relation with productivity, respiratory rate (RR), and rectal temperature (RT) in heat stressed dairy cows. The study was conducted during the summer using 48 Holstein cows. Results showed that the ambient temperature and relative humidity ranged between 20°C and 30°C and 49% to 84% during the study. Resveratrol treatment increased plasma total antioxidant capacity up to 12% in parity 2 cows. Regression analysis indicated that plasma total antioxidant capacity had a positive relationship with changes in milk yield in resveratrol cows, and the increase of 1 mM plasma total antioxidant capacity was associated with an increase of 8.5 kg of milk yield. Resveratrol increased plasma total antioxidant capacity and mitigated oxidative stress and production loss in heat-stressed dairy cows. RES = resveratrol; TEAC = trolox equivalent antioxidant capacity.

Despite progress in management and housing facilities, heat stress continues to impair productivity, welfare, and health in animal agriculture. Traditionally, factors such as reduced nutrient supply have been associated with lower productivity in dairy cattle exposed to heat stress. During heat stress and compared with control multiparous cows, their counterparts consuming 14% or 28% more protein increased catabolic metabolism instead of mammary synthesis of ECM and proteins (Kaufman et al., 2017). Subsequently, we have recently demonstrated that heat stress losses in milk production in dairy cows and growth performance in dairy calves occurred in association with the activation of the immune response (Kaufman et al., 2021; Ríus et al., 2022; Yu et al., 2024, 2025).

To counter heat stress pathophysiology, dietary supplementation with *Aspergillus oryzae* extract rich in antioxidant compounds increased yields of milk, ECM, fat, and protein by 9% to 13%, and increased nutrient allocation toward lactation by 4% (Kaufman et al., 2021). Among the metabolites synthesized by *Aspergillus oryzae*, kojic acid and its derivatives have been shown to exhibit a variety of biological functions, including antioxidant and anti-inflammatory effects (Zilles et al., 2022). Our previous research showed that feeding an *Aspergillus oryzae* extract reduced concentrations of proinflammatory biomarkers in plasma of heat-stressed cows (Kaufman et al., 2021) and calves (Ríus et al., 2022) implying a reduction in systemic inflammation. In essence, reducing proinflammatory biomarkers suggests breaking the inflammatory cycle. It is possible, however, that heat stress impairment in nutrient utilization is also linked to changes in oxidative stress implied by the positive effects of *Aspergillus oryzae* extract administration (e.g., antioxidant effect) and its association with a robust downregulation of metallothionein genes in fruit flies (Kaufman et al., 2021).

Hyperthermia promotes oxidative stress by triggering the production of oxidants to an extent where the oxidants exceed the neutralizing capacity of the antioxidant defense system in dairy cows (Guo et al., 2021; Li et al., 2021). The production of oxidants links inflammation and milk synthesis such that heat stress–increased oxidative stress parallels apoptosis of mammary cells and, ultimately, a decline in milk yield (Zachut et al., 2017; Safa et al., 2019; Guo et al., 2021). Radical preventive antioxidants constitute the first line of defense. These antioxidants are enzymes that act very quickly to suppress molecules with the potential of developing into free radicals (i.e., chain initiation) or any free radical with the ability to trigger production of oxidants (i.e., chain propagation). For example, superoxide dismutase (**SOD**) converts the superoxide radical into oxygen and hydrogen peroxide. Metal ion binding enzymes (e.g., metallothionein) chelate iron and copper, preventing them from free radical formation. Radical scavengers, the second line of defense, neutralize active radicals to inhibit chain initiation and break chain propagation reactions. For example, kojic acid scavenges free radicals by donating electrons, reducing them to a harmless molecule.

Phytochemicals such as polyphenols (e.g., resveratrol) found in dietary ingredients reduce oxidative stress and have therapeutic effects across species (Frémont, 2000; Meng et al., 2023). Resveratrol reduces oxidative stress by scavenging free radicals like the mechanism described for kojic acid (i.e., nonenzymatic action) and, to a lower extent, by increasing the activity of antioxidant enzymes such as SOD and glutathione peroxidase (i.e., enzymatic action; Hussain et al., 2016; Xia et al., 2017). A subclinical trial to test the therapeutic effect of resveratrol in heat-stressed rats showed a decrease in the plasma concentration of malondialdehyde (**MDA**), a well-known biomarker of lipid peroxidation (Cheng et al., 2019). In accordance with this, Das (2011) found that resveratrol pretreatment decreased the development of heat stress–associated oxidative stress in rats. The therapeutic role of resveratrol associated with elevated plasma SOD activity and antioxidant capacity, as well as reduced plasma MDA concentrations, was demonstrated in chickens (Liu et al., 2014), sheep (González-Garzón et al., 2023), and humans (Pintea et al., 2011).

Even though the therapeutic effects of resveratrol have been studied before, the use of resveratrol to reduce heat stress–associated oxidative stress in dairy cattle has not been investigated. Studying the antioxidant role of resveratrol can offer new insights into its use as a nutritional strategy to mitigate oxidative stress in lactating dairy cows. Therefore, we hypothesized that supplementing resveratrol would improve the antioxidant capacity and reduce heat stress–induced oxidative stress in lactating dairy cows.

This study was conducted with the approval of the Institutional Animal Care and Use Committee of the University of Tennessee (protocol no. 2918–0522). A total of 48 cows were housed in 1 of 2 pens (24 per pen) in a deep sand–bedded freestall barn of East Tennessee Ag-Research and Education Center-Little River Animal and Environmental Unit (ETREC-LRAEU, Walland, TN) during the summer months in 2022. Cows were randomly assigned into 1 of the 2 treatment groups: control diet (**CON**; no resveratrol supplement) and resveratrol diet (**RES**; 500 mg/d of resveratrol supplementation; 8.9 g of product, Thrive Animal Health, LLC). Within treatment group, 30 Holstein cows averaging (±SD) 127 ± 33 DIM, 48.9 ± 8.9 kg/d milk yield, and 2.1 ± 1.0 parity were used in this study (CON = 15 cows and RES = 15 cows). Resveratrol dosage was determined using the literature and manufacturer's recommendation and was reported in our previous study (Nair et al., 2025). All animals were acclimatized to their respective treatments for 2 wk before the start of the trial. The trial was imposed for 3 wk during which the heat-abatement tools such as fans and sprinklers were disabled in both pens for animals to experience natural summer heat stress. Animals were offered a common TMR twice daily and the details of diet ingredients and other physiological measurements are described in a companion paper (Nair et al., 2025). Blood samples were collected once a week during the acclimatization and treatment periods from coccygeal vessels using 10-mL Vacutainer tubes (Becton Dickinson and Co., Franklin Lakes, NJ). Blood samples were centrifuged at 1,200 × *g* for 10 min at 4°C and plasma was harvested, aliquoted, and stored at −80°C until oxidative stress assays were conducted. Respiratory rate (**RR**; breaths/min) was recorded along with rectal temperature (**RT**) at 0730, 1200, and 1830 h every day during the trial. Rectal temperature was recorded using a calibrated GLA M700 digital thermometer. Cows were milked at 0600 and 1700 h in a herringbone parlor using electronic milk meters (BouMatic, Magnum M series GT2).

Loggers (HOBO, Onset Computer Corp., Bourne, MA) were installed at cow height in both pens to measure the ambient temperature and relative humidity, every 5 min throughout the study.

Plasma concentrations of total antioxidant capacity and biomarkers of oxidations such as MDA and SOD were determined according to the manufacturer's instructions provided with the commercial assay kits (catalog #709001 [total antioxidant capacity], #10009055 [MDA], and #706002 [SOD]; Cayman Chemicals, Ann Arbor, MA). Complex antioxidant systems in biological fluids, both enzymatic and nonenzymatic, reduce reactive oxygen species (**ROS**) concentration and ROS-induced damage. Enzymatically, SOD scavenges superoxide radicals to produce hydrogen peroxide, which is then reduced by catalase or glutathione peroxidase. Nonenzymatic antioxidants include glutathione, vitamins E and A, ferritin, and ceruloplasmin. Together, enzymatic and nonenzymatic antioxidative factors comprise the total antioxidant capacity of a system and work in an integrated way to reduce oxidative stress. It is difficult and costly to accurately quantify each individual component of the antioxidant system. As a result, the total antioxidant capacity method considers the interaction of all antioxidants present in a sample. Briefly, the method measures the capacity of a sample to inhibit the 2,2′-azino-di-3-ethylbenzthiazoline-6-sulfonic acid (**ABTS**) radical (**ABTS+**) compared with a standard antioxidant reference (trolox, a synthetic water-soluble tocopherol analog). The ABTS+ radical is generated by chemical reaction of potassium persulfate with ABTS. A known volume of trolox at standard concentration results in a similar reduction of the radical ABTS+. This ABTS+ radical is blue and reacts with most antioxidants. During this reaction, the blue ABTS+ radical cation is converted back to its colorless neutral form. The calibration curve is prepared for a concentration range of 0 to 0.495 m*M* trolox. The absorbance obtained for plasma samples is interpolated to calculate concentration in m*M* of trolox equivalent antioxidant capacity (**TEAC**). A 96-well standard microplate was used to run every sample in duplicates using a spectrophotometer at 750 nm (Biotek, Santa Clara, CA). The intra- and interassay CV ranged from 2% to 8%.

During oxidative stress, peroxides derived from catabolism of PUFA form a series of complex compounds, which include reactive carbonyl compounds (e.g., MDA). Malondialdehyde reacts with thio-barbituric acid producing a red pigment that can be quantified by spectrophotometry in the form of thio-barbituric acid reactive substances (**TBARS**). The measurement of TBARS was established as a method for monitoring the formation of fatty acids peroxides during oxidative stress. Briefly, the MDA–thio-barbituric acid (**TBA**) adduct formed by the reaction of MDA with TBA under high temperature and acidic conditions was measured colorimetrically using spectrophotometry at 530 nm and concentrations ranged between 0.625 and 50 μ*M* of MDA equivalents. The intra- and interassay CV was between 2% and 6%.

The tetrazolium salt detection method was used in determining the activity of SOD, and the readings were taken at a wavelength of 440 nm. The intra- and interassay CV were between 5% and 9%. Pro-oxidant–antioxidant balance (**PAB**; Alamdari et al., 2007) is determined from concentration of MDA and TEAC and expressed in the arbitrary unit HK as

PAB = MDA/TEAC.

Data collected before treatments were used as a covariate in the statistical analysis. The TEAC, SOD, and MDA data were analyzed using a mixed model procedure with repeated measurements in SAS (version 9.4; SAS Institute Inc., Cary, NC). The effects of treatment, week, and parity and their interactions were included as fixed effects and animal nested with treatment was included as a random effect. The effect of week was included as a repeated measure. Normality and homoscedasticity of the studentized residuals were analyzed using the Shapiro–Wilk test. A suitable covariance structure was chosen based on the Akaike information criterion score. Log-transformation was performed for SOD analysis and nontransformed data were reported as LSM. All other analyses were performed without data transformation. The statistical model used for analysis is shown below:

Y_ijkl_ = μ+ T_i_ +D_j_+ P_k_ + A_l_ + β(χ)_ijkl_ + interactions + ε_ijkl_,

where Y_ijkl_ is the dependent variable; μ is the the overall mean; T_i_ is the fixed effect of the ith treatment (i = CON and RES); D_j_ is the fixed effect of jth week as repeated measures; P_k_ is the fixed effect of kth parity (1, 2, and ≥ 3), A_l_ is the random effect of the lth animal; β(χ)_ijkl_ is the covariate effect; and ε_ijkl_ is the error. Significant difference was declared at *P* < 0.05 and a trend was declared when 0.10 < *P* ≥ 0.05. Regression analyses were conducted using milk yield against TEAC and RR or RT against TEAC using the Reg procedure in SAS as described in Nair et al. (2025), and significance was declared for any *P*-value ≤ 0.05.

The hourly average of ambient temperature ranged between 20°C and 30°C. The hourly average of relative humidity ranged between 49% and 84% (Figure 1). Overall, compared with CON, RES increased the plasma TEAC concentrations (1.10 vs. 1.04 m*M*; *P* = 0.01). There was a treatment × parity interaction (*P* = 0.02) such that the plasma TEAC concentration was 12% higher in parity 2 RES group cows compared with the respective CON group (1.14 vs. 1.02 ± 0.01 m*M*). Compared with CON, RES cows in parity 1 and 3 or more had a numerically higher plasma TEAC concentration (Table 1). Plasma TEAC concentrations were similar or slightly higher than those reported previously (Ghiselli et al., 2000). Treatments did not affect (*P* = 0.17) plasma MDA concentrations in heat-stressed cows (Table 1). Treatments did not affect (*P* = 0.89) plasma SOD concentrations in heat-stressed cows (Table 1). Treatments tended to affect PAB (treatment × week interaction, *P* = 0.07). Treatment RES cows had a numerical lower PAB during wk 2 and 3 (Figure 2A).

**Figure 1:**
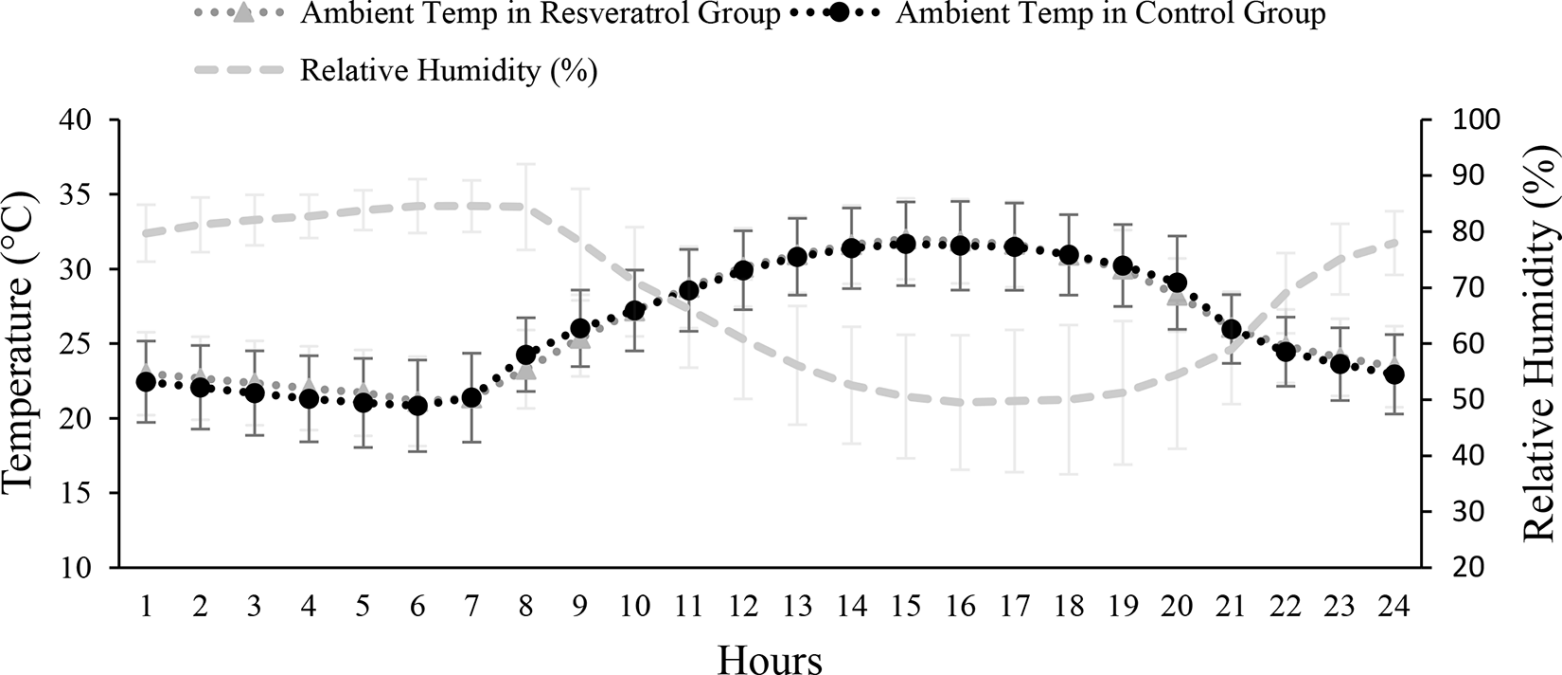
Arithmetic mean of ambient temperature (temp; °C) for both resveratrol (RES) and control (CON) pens with the mean of relative humidity (%) for a day. Each point is an average for an hour. Data presented as mean ± SEM.

**Table 1:** The plasma concentration of antioxidants in heat-stressed lactating Holstein cows in both resveratrol and control groups

Parameter^2^	Parity 1		Parity 2		Parity ≥3		SEM	Effect^1^ (*P-*value)		
	CON	RES	CON	RES	CON	RES		T	Parity	T × Parity
TEAC (m*M*)	1.04^b^	1.06^b^	1.02^b^	1.14^a^	1.06^b^	1.09^b^	0.011	0.005	0.210	0.025
MDA (μ*M*)	11.59	12.00	11.91	12.50	11.56	12.10	0.264	0.175	0.674	0.976
SOD (U/mL)	1.36	1.36	1.38	1.30	2.04	1.45	0.211	0.888	0.489	0.657
PAB (HK)	12.68	12.36	11.93	11.18	11.34	11.65	0.298	0.525	0.088	0.544

a,b Different superscripts within a row denote a significant difference (*P* < 0.05).

1 T = treatment (CON, control RES, resveratrol); T × Parity = treatment and parity interaction.

2 TEAC = total antioxidant capacity (measured as trolox equivalent antioxidant capacity); MDA = malondialdehyde; SOD = super oxide dismutase; PAB = pro-oxidant–antioxidant balance = MDA/TEAC.

**Figure 2:**
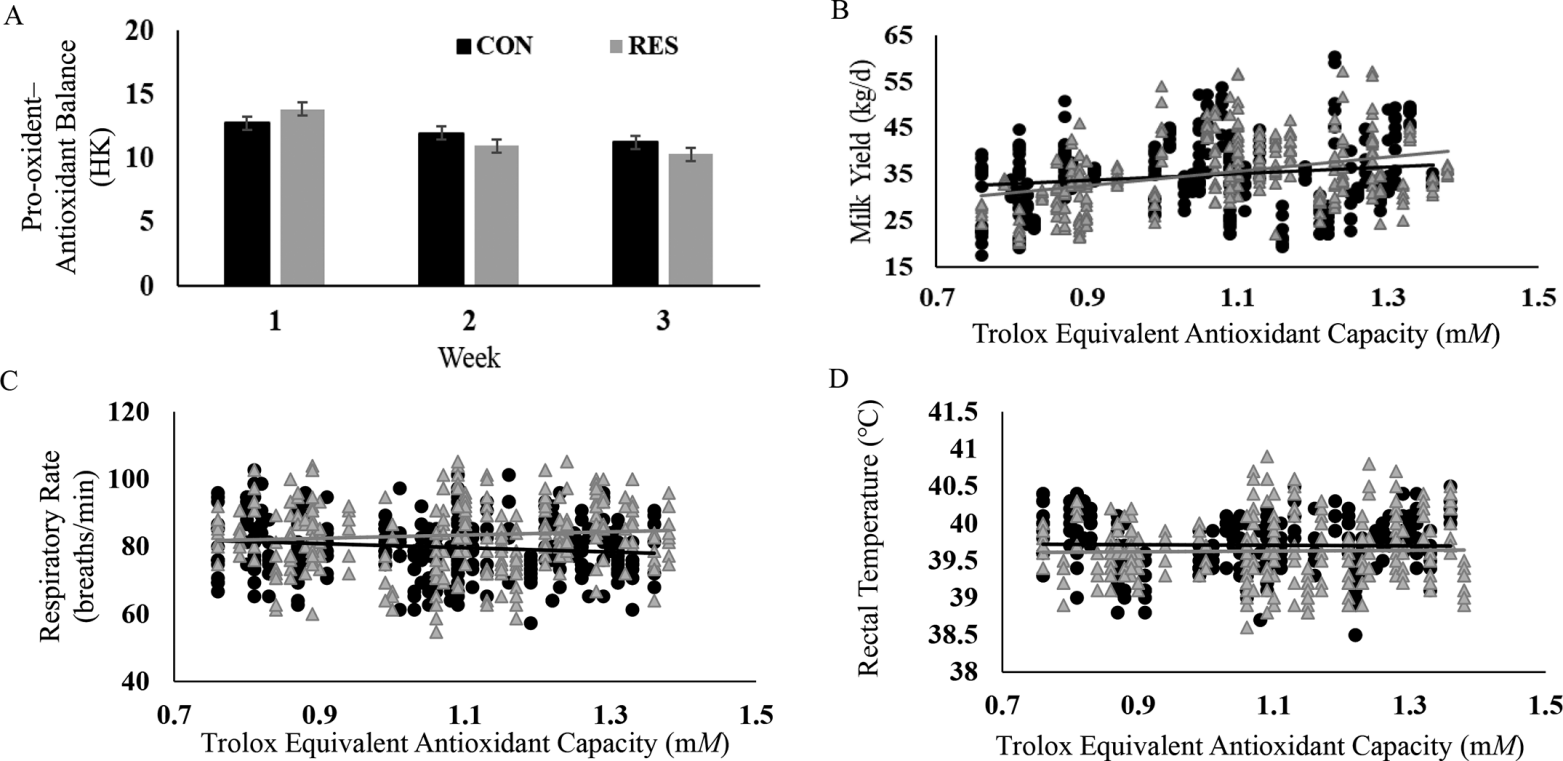
(A) The RES treatment tended to change the pro-oxidant–antioxidant balance (PAB, treatment × week, *P* = 0.07; derived in arbitrary unit HK). (B) Milk yield regressed against total antioxidant capacity (measured as TEAC). The linear relationship of milk yield with total antioxidant capacity for CON and RES groups are shown as y = 27.32 + 7.10x (R^2^ = 0.02; *P* = 0.004) and y = 18.55+15.62x (R^2^ = 0.13; *P* < 0.001), respectively. (C) Average RR regressed against total antioxidant capacity (measured as TEAC). The linear relationship of average RR with total antioxidant capacity for control (CON) and resveratrol (RES) groups are respectively shown as y = 86.50 − 6.23x (R^2^ = 0.01; *P* = 0.02) and y = 78.01 + 4.80x (R^2^ = 0.007; *P* = 0.13), respectively. (D) Average RT regressed against total antioxidant capacity (measured as TEAC) showed no relation. Control (CON) and resveratrol (RES) groups were shown as y = 39.74 − 0.03x (R^2^ = 0.0004; *P* = 0.21) and y = 39.57 + 0.05x (R^2^ = 0.0003; *P* = 0.73), respectively.

Simple linear regression analyses revealed relationships between milk yield and average RR with plasma TEAC concentration (Figures 2B–C). Although weak, a positive relationship existed between plasma TEAC concentration and milk yield irrespective of treatments (CON, R^2^ = 0.02, *P* < 0.01 and RES, R^2^ = 0.13, *P* < 0.01). Compared with CON cows, RES cows showed greater milk production when plasma TEAC concentration increased from 0.7 to 1.4 m*M*. There was an increase of 7.10 and 15.6 kg of milk yield per unit of plasma TEAC concentration in CON and RES treatments, respectively. A weak relationship existed between plasma TEAC concentration and RR in CON cows only (R^2^ = 0.01, *P* = 0.02 and RES, R^2^ = 0.007, *P* = 0.13). With a unit increase in plasma TEAC concentration, CON cows decreased RR by 6 breaths/min. There was no relationship between RT and plasma TEAC (Figure 2D).

The goal of this study was to determine the effect of resveratrol supplementation in alleviating heat stress–associated oxidative stress in lactating dairy cows. In the current study, the hourly ambient temperature was ≥25°C and relative humidity was >50% for the greatest part of the treatment period, which indicated mild to severe heat stress conditions in the barn. According to Berman et al. (1985), the upper critical temperature for dairy cattle is 25°C, confirming that the environmental conditions were sufficient to elicit a heat stress response in dairy cows. The physiological response of cows and environmental aspects of the current study were published elsewhere (Nair et al., 2025).

Our results revealed that resveratrol supplementation improved plasma TEAC concentration with a distinguishable 12% increase in parity 2 cows. Our results agreed with the findings of Zhou et al. (2023) where supplementing resveratrol at 400 mg/kg of diet fed improved the plasma antioxidant capacity in heat-stressed ducks. Similarly, resveratrol supplementation at 150 mg/kg of diet fed was found to improve serum and muscle concentrations of antioxidant capacity in heat-stressed broilers (Yang et al., 2021). The significance of measuring plasma TEAC concentration has been widely accepted in drawing quantitative conclusions concerning enzymatic and nonenzymatic antioxidants concentrations in body fluids (Silvestrini et al., 2023). It offers a broader yet integrative assessment of the total (i.e., enzymatic and nonenzymatic) antioxidant status of the animal.

Consistent with our observation of improved plasma TEAC concentration in RES-fed parity 2 cows, recent research reported an increase in glutathione peroxidase concentration in older mares (>12 years old; Rosa Filho et al., 2025). Although it was not measured in our study and compared with parity 1, it is possible that RES parity 2 cows increased glutathione peroxidase activity and, therefore, an associated increased plasma TEAC concentration. A study conducted in lactating ewes found that milk total antioxidant capacity increased with lactation number in mature ewes compared with first-lactation ewes (Stocco et al., 2024). In our study the supplementation with 500 mg of resveratrol reduced RT in parity 1 and 2 cows compared with parity ≥3 cows (discussed in Nair et al., 2025), possibly indicating that the treatment was not effective in cows with higher metabolism (i.e., parity ≥3 cows). In agreement with our previous findings, a recent study in lactating dairy cows indicated that supplementation with resveratrol at 8 mg/kg of BW per day lowered RT and increased milk yield and DMI when compared with a 4 mg/kg BW of resveratrol treatment (Ahvanooei et al., 2025). Collectively, it is possible that resveratrol supplementation at 500 mg was enough to improve plasma antioxidant capacity in parity 2 cows but not in parity ≥3 cows, which potentially had a higher production of oxidants.

In our study, we did not observe changes in plasma SOD activity, even though RES treatment increased TEAC concentration. In agreement with this result, resveratrol supplementation at 92 mg/kg of BW increased antioxidant capacity but it did not change SOD activity in the jejunum epithelium of broilers exposed to heat stress (Wang et al., 2021). Conversely, resveratrol supplementation (587 mg/kg of BW) increased plasma SOD activity in black-boned chickens exposed to heat stress (Liu et al., 2014). Clearly, the effect of resveratrol in SOD activity appears to be dose, tissue, and species specific. In the present study, our data indicate that resveratrol improved plasma antioxidant capacity by increasing the nonenzymatic part of the antioxidant defense system. In comparison with previous work supplementing a nonenzymatic antioxidant-containing product, we showed that the pathological phenotypes in fruit flies resulting from heat stress were markedly mitigated when feeding the product (Kaufman et al., 2021). Interestingly, the antioxidant-fed flies downregulated gene expression of antioxidant enzymes (i.e., metallothionein B, C, and D). However, this finding was a secondary effect and a consequence of higher status of total antioxidant capacity, and not a direct target of the mode of action of the antioxidant product. Collectively from our study, it is possible that the resveratrol dose did not induce an SOD-mediated antioxidant response. This highlights the importance of characterization of enzymatic and nonenzymatic components of the antioxidant defense system when studying oxidative status of heat-stressed cows treated with different concentrations of resveratrol.

Our results revealed no changes in plasma MDA concentration. In comparison, our results were slightly lower than the plasma MDA concentrations reported in periparturient dairy cows that are known to display oxidative stress (Castillo et al., 2006). The latter suggests that hyperthermia-produced oxidative stress in our study could have been milder relative to that observed in periparturient cows. Malondialdehyde is a byproduct of lipid peroxidation that is produced when free radicals attack carbon-carbon linkages in PUFA (Vlachogianni et al., 2015). In agreement with our study, resveratrol supplementation (46 mg/kg of BW) did not affect plasma MDA concentration in heat-stressed yellow feather broilers. However, there was a linear reduction in plasma MDA when resveratrol concentration increased to 69 and 115 mg/kg of BW (He et al., 2019). The contradictory nature of previous and current work could be due to underlying factors, including animal age, supplement dosage, treatment length, and the mild to severe nature of heat stress, and thus warrants further investigation.

The regression analysis revealed that the plasma TEAC concentration explained a small fraction of the change in milk yield. Indeed, in CON cows, the model explained 2% of milk yield reported, indicating that plasma TEAC concentrations weakly relate to milk yield. High plasma TEAC concentrations were probably paralleled with comparable production of oxidants and an elevated plane of oxidative stress, hence the weak association between TEAC with milk production. However, the increase in plasma TEAC in RES treatment cows accounted for a 13% increase in milk production. This result indicated that resveratrol supplementation improvement of milk production (discussed in Nair et al. 2025) was explained in part by the increase in plasma TEAC. It is possible that high plasma TEAC concentrations in RES cows occurred in parallel with elevated scavenger activity and lower plane of oxidative stress. The regression analysis revealed a quantitative response concerning the changes in plasma TEAC due to a resveratrol treatment in milk synthesis. Collectively, the implication is that resveratrol, a nonenzymatic antioxidant, may scavenge reactive oxidants and reduce hyperthermia-associated oxidative stress.

In conclusion, resveratrol supplementation enhanced plasma total antioxidant capacity in heat-stressed dairy cows. The regression analysis revealed that the increase in plasma total antioxidant capacity was linked with a 13% increase in milk production in resveratrol-treated cows. The improvement in antioxidant status may have improved energy utilization, which may have been translated into supporting milk synthesis. Further investigation is needed to check the direct relationship between the regression factors. Resveratrol could be used as a feed additive to reduce the effect of heat stress on commercial dairy farms.
